# Prevalence and Genotype Distribution of *Giardia duodenalis* in Rabbits in Shandong Province, Eastern China

**DOI:** 10.1155/2020/4714735

**Published:** 2020-02-17

**Authors:** Tao-Shan Li, Yang Zou, Jun-Jie Peng, Li-Qun Wang, Hai-Sheng Zhang, Wei Cong, Xing-Quan Zhu, Xiao-Lin Sun

**Affiliations:** ^1^College of Veterinary Medicine, Gansu Agricultural University, Lanzhou, Gansu 730070, China; ^2^State Key Laboratory of Veterinary Etiological Biology, Key Laboratory of Veterinary Parasitology of Gansu Province, Lanzhou Veterinary Research Institute, Chinese Academy of Agricultural Sciences, Lanzhou, Gansu 730046, China; ^3^Marine College, Shandong University at Weihai, Weihai, Shandong 250100, China; ^4^Jiangsu Co-Innovation Center for the Prevention and Control of Important Animal Infectious Diseases and Zoonoses, Yangzhou University College of Veterinary Medicine, Yangzhou, Jiangsu 225009, China

## Abstract

*Giardia duodenalis* is a zoonotic enteric parasite that can infect humans and a number of animal species including rabbits with a worldwide distribution. Infection with *G. duodenalis* can cause serious public health problems and significant economic losses to animal husbandry. So accurate understanding of the prevalence and genotype distribution of *G. duodenalis* in rabbits is necessary. In the present study, a total of 616 fecal samples were collected from rabbits in Shandong province, eastern China, and examined in *G. duodenalis* prevalence and genotypes by nested PCR amplification of *β*-giardin (bg), glutamate dehydrogenase (gdh), and triosephosphate isomerase (tpi) gene loci of *G. duodenalis*. Sixty-nine (11.2%) of the examined rabbit fecal samples were *G. duodenalis-*positive. Of them, the prevalence of *G. duodenalis* is 8.4% (41/490) in Rizhao city and 22.2% (28/126) in Weihai city. Breeds, region, and feeding modes were highly correlated with *G. duodenalis* infection in rabbits. Moreover, three genotypes (assemblages A, B, and E) were identified in rabbits at three gene loci, and the assemblage E was the dominant genotype, while the assemblage A was reported in rabbits in China for the first time. It is noticeable that two rabbits were found to be infected with two different *G. duodenalis* assemblages (assemblages A and E, assemblages B and E, respectively). These findings enrich the genotype distribution of *G. duodenalis* in rabbits and provide baseline data for preventing and controlling *G. duodenalis* infection in rabbits in eastern China.

## 1. Introduction

*Giardia duodenalis* (syn. *Giardia intestinalis*, *Giardia lamblia*) is a common gastrointestinal protozoon that causes enteric disease in a variety of animal species and humans [1–4]. More than 40 species of animals have been reported to be infected with *G. duodenalis* over the world [2, 5]. Humans can be infected through ingesting water and food contaminated with *G. duodenalis* cysts [6]. The clinical symptoms of giardiasis are diarrhea, dehydration, abdominal pain, nausea, vomiting, and weight loss [2, 6, 7]. Giardiasis caused by *G. duodenalis* also has been recognized as an important zoonotic disease for both public and animal health [2, 7, 8].

Eight assemblages have been identified in *G. duodenalis*, including assemblages A–H [2, 5]. The structure of eight assemblages is similar, but the genotypes are distinct [8]. Among assemblages A–H, both of the assemblages A and B are usually identified in various mammals, including humans [9], while the remaining assemblages mainly occur in the relatively specific groups of animals [10, 11].

Rabbits are one of the most important economic animals in China, and the consumption of meat and fur accounts for a large part of China's economy [12]. However, rabbits are susceptible to many pathogens [5, 13, 14], including *G. duodenalis* [15–20], which can cause significant economic losses to the rabbit breeding industry. There are limited reports about *G. duodenalis* prevalence in rabbits in China [15, 18–20]; therefore, the objective of the present study was to investigate the prevalence and genotype distribution of *G. duodenalis* in rabbits in Shandong province, eastern China.

## 2. Materials and Methods

### 2.1. Collection of Fecal Samples

A total of 616 rabbit fecal samples (490 from Rizhao city, 126 from Weihai city) were collected from Shandong province, eastern China. The fecal samples were composed of 207 New Zealand white rabbits, 188 long-haired rabbits, and 221 Tolai hares. Each fresh fecal sample was collected with gloves and placed into a box with ice, then marked with the breed, age, and region, respectively, and transported to the laboratory. All of the fecal samples were stored at −20°C for further DNA extraction.

### 2.2. Genomic DNA Extraction

Each rabbit fecal sample was washed with distilled water, uniformly stirred, and the residue was filtered through a 60-mesh sieve (0.23 × 0.23 mm). The filtrate was centrifuged at 4000*g* for 5 min in a centrifuge, the filtrate was discarded, and the precipitate was retained; then approximately 300 mg of each precipitate of fecal sample was used to extract genomic DNA using the commercial E.Z.N.A.R® Stool DNA Kit (Omega Bio-Tek Inc., Norcross, GA, USA), following the manufacturer's recommendations. The extracted genomic DNA was stored at −20°C for further PCR amplification.

### 2.3. Nested PCR Amplification

The prevalence and genotypes of *G. duodenalis* were examined by nested PCR amplification of the bg, gdh, and tpi gene loci as described previously [2, 15]. The primers and corresponding annealing temperature are shown in Table 1. Positive and negative controls were included in each PCR reaction. The secondary PCR products were electrophoresed in 1% (w/v) agarose gels containing ethidium bromide.

### 2.4. Sequence Analysis and Phylogeny

All of the positive secondary PCR products were sent to Tsingke Biotechnology Technology Company (Xi'an city, China) for two-directional sequencing. The obtained sequences were compared with reference sequences in GenBank database (http://www.ncbi.nlm.nih.gov/BLAST/) and edited using computer Clustal X 1.83 [21]. The phylogenetic tree was constructed using the Neighbor-Joining [NJ] analysis in MEGA7 (https://www.megasoftware.net/), the Kimura 2-parameter model was selected, and 1,000 bootstrap replicates were calculated.

### 2.5. Statistical Analysis

The relationship between the *G. duodenalis* prevalence in rabbits with different variables such as breed, region, age, and feeding mode was analyzed by the Chi-square (*χ*^2^) test. The 95% confidence intervals (CIs) and odds ratios (ORs) were estimated by SPSS version 24.0 (SPSS Inc., Chicago, IL, USA). If the statistical result*P* < 0.05, the difference was considered statistically significant.

## 3. Results and Discussion

### 3.1. The Prevalence of *G. duodenalis* in Rabbits

In the present study, 69 (11.2%, 95% CI: 8.71–13.69) of the examined 616 rabbit fecal samples were positive for *G. duodenalis* by nested PCR amplification of the bg, gdh, and tpi loci (Table 2). Among different breeds, the highest prevalence was 23.1% (95% CI: 17.52–28.63) in Tolai hares, followed by 7.3% (95% CI: 3.72–10.78) in New Zealand white rabbits and 1.6% (95% CI: 0.19–3.39) in long-haired rabbits, and the difference was considered significant (*χ*^2^ = 50.03, d*f* = 2, *P* < 0.01). Moreover, the *G. duodenalis* prevalence in rabbits from Rizhao city (8.4%, 95% CI: 5.92–10.82) was significantly lower than that from Weihai city (22.2%, 95% CI: 14.96–29.48) (*χ*^2^ = 19.34, d*f* = 1, *P* < 0.01). Rabbits raised outdoors had a significantly higher *G. duodenalis* prevalence (23.1%) than that raised indoors (4.6%) (*χ*^2^ = 48.87, d*f* = 1, *P* < 0.01) (Table 2). Rabbits that are less than 6 months had a slightly lower *G. duodenalis* prevalence of 9.4% (95% CI: 5.93–12.77) than rabbits of more than 6 months old (12.7%, 95% CI: 9.17–16.27) (Table 2). The *G. duodenalis* prevalence in male rabbits (11.5%) (95% CI: 7.70–15.33) was slightly higher (*χ*^2^ = 0.05, d*f* = 1, *P*=0.82) than that in female rabbits (11.0%) (95% CI: 7.67–14.22) (Table 2).

In the present study, the overall *G. duodenalis* prevalence in rabbits was 11.2% (69/616), which was higher than that in rabbits in Henan province (8.4%, 80/955) [15], Jilin province and Liaoning province (9.86%, 42/426) [18], Heilongjiang province (7.41%, 28/378) [20], Xinjiang province (1.9%, 6/321) [19] in China, and in Europe (7.6%, 40/528) [16] and Melbourne, Australia (1.03%, 1/97) [22], but lower than that in rabbits in Ecuador (20.0%, 4/20) [17]. The different *G. duodenalis* prevalence in rabbits may be caused by many factors such as geographical ecological environment, detection methods, sample size, and individual health status [23].

### 3.2. Molecular Characterization of *G. duodenalis* Isolates

Two *G. duodenalis* assemblages (assemblages B and E) have been reported in rabbits in China [15, 18–20]. In the present study, 616 rabbit fecal samples were used to identify *G. duodenalis* genotypes by nested PCR targeting the bg, gdh, and tpi genes. The results showed that 53 bg-positive samples and 39 gdh-positive samples were identified as assemblage E; two (2.9%, 2/69) tpi-positive samples and one (1.5%, 1/69) tpi-positive sample were identified as assemblage B and assemblage A, respectively. Interestingly, the assemblage E (98.6%, 68/69) was the dominant genotype in rabbits in the present study, which was different from the previous studies [18, 19]. Moreover, two mixed *G. duodenalis* assemblages (assemblages A and E, assemblages B and E) were identified in rabbits, and the assemblage A was firstly detected in rabbits in China in the present study. The *G. duodenalis* assemblage A was also reported in humans [9, 22]. This finding suggests that the rabbits may be a potential source of human infection with *G. duodenalis.*

### 3.3. Phylogenetic Analysis of *G. duodenalis* Isolates in Rabbits

**Figure 1:**
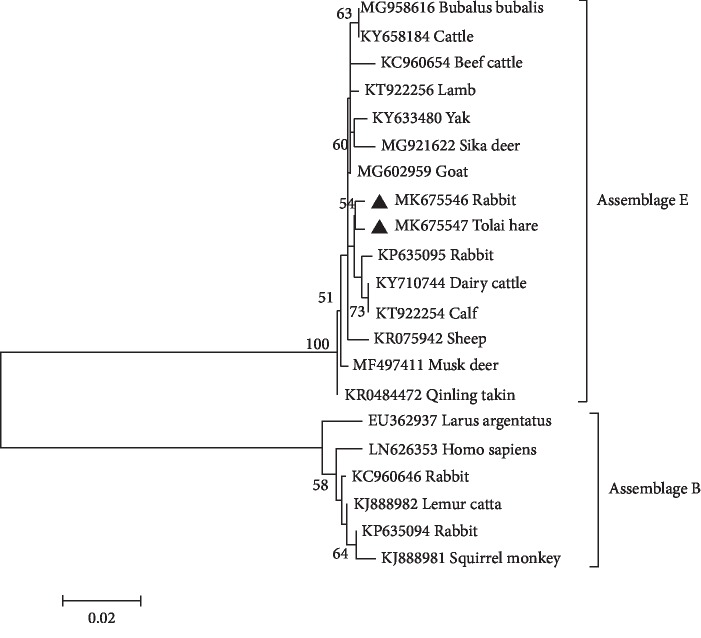
The phylogenetic relationships among *G. duodenalis* isolates. The numbers on the branches represent percent bootstrapping values from 1000 replicates, with values of more than 50% shown in the tree. The genotypes of *G. duodenalis* which were identified at gdh gene locus in the present study are marked by filled triangles.

**Figure 2:**
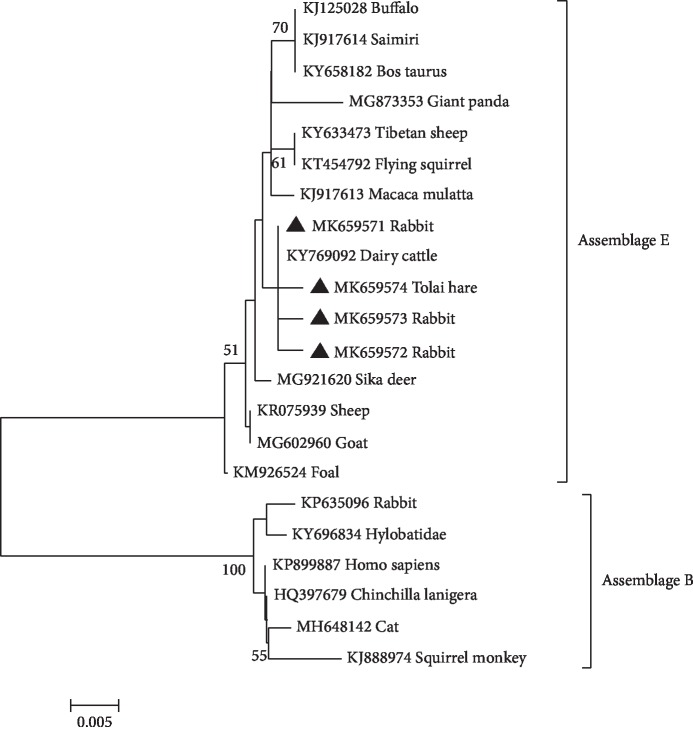
The phylogenetic relationships among *G. duodenalis* isolates inferred by a Neighbor-Joining (NJ) algorithm using a Kimura two-parameter analysis (1000 replicates) based on the bg gene sequences. The genotypes of *G. duodenalis* which were identified at bg gene locus in the present study are marked by filled triangles.

To further elucidate the genetic relationship of *G. duodenalis* assemblages in rabbits, we aligned the obtained sequences with reference sequences in GenBank by Clustal X 1.83, which were used for phylogenetic analyses (Figures 1 and 2). The phylogenetic analyses showed that the *G. duodenalis* assemblage E in rabbits and dairy cattle was distributed on one branch, representing a closer genetic relationship (Figure 2). These findings indicated a possibility of spreading *G. duodenalis* between rabbits and dairy cattle. Moreover, the single nucleotide polymorphisms (SNPs) existed in bg sequences and gdh sequences in this study (Table 3) by comparing the obtained *G. duodenalis* sequences in the present study with corresponding sequences in the GenBank database. These findings indicated that various *G. duodenalis* types are distributed in rabbits. These findings enriched the genetic diversity of *G. duodenalis* in rabbits and other animals.

## 4. Conclusions

The present study revealed a higher (11.2%) *G. duodenalis* prevalence in rabbits in Shandong province, eastern China. Different feeding methods, breeds, and regions were highly correlated with *G. duodenalis* prevalence in rabbits (*P<0.05*). Three *G. duodenalis* assemblages (assemblage A, assemblage B, and assemblage E) were identified in rabbits, assemblage A was reported in rabbits in China for the first time, while assemblage E was the dominant assemblage. These findings not only enriched the genotype distribution of *G. duodenalis* in rabbits but also have an implication for better control of *G. duodenalis* in rabbits. Further studies are necessary to examine the infection status and genotype distribution of *G. duodenalis* in rabbits in other areas of the country.

## Figures and Tables

**Table 1:** Nest PCR primers and annealing temperatures used in this study.

Gene	Primer	Sequence (5′-3′)	Annealing temperature (°C)	Length (bp)	Reference
bg	F1	AAGCCCGACGACCTCACCCGCAGTGC	55		[2]
	R1	GAGGCCGCCCTGGATCTTCGAGACGAC			
	F2	GAACGAACGAGATCGAGGTCCG	55	511	
	R2	CTCGACGAGCTTCGTGTT			

gdh	F1	TTCCGTRTYCAGTACAACTC	50		[2]
	R1	ACCTCGTTCTGRGTGGCGCA			
	F2	ATGACYGAGCTYCAGAGGCACGT	65	530	
	R2	GTGGCGCARGGCATGATGCA			

tpi	F1	AAATIATGCCTGCTCGTCG	55		[2]
	R1	CAAACCTTITCCGCAAACC			
	F2	CCCTTCATCGGIGGTAACTT	55	530	
	R2	GTGGCCACCACICCCGTGCC			

**Table 2:** Prevalence and risk factors of *Giardia duodenalis* infection in rabbits.

Factor	Category	No. tested	No. positive	% (95% CI)	*P* value	OR (95% CI)
Breeds	Long-haired rabbit	188	3	1.6 (0.19–3.39)	0.01	Reference
	New Zealand white rabbit	207	15	7.3 (3.72–10.78)		4.82 (1.37–16.92)
	Tolai hare	221	51	23.1 (17.52–28.63)		18.50 (5.67–60.38)

Region	Rizhao	490	41	8.4 (5.92–10.82)	0.01	Reference
	Weihai	126	28	22.2 (14.96–29.48)		3.13 (1.85–5.30)

Feeding	Indoors	395	18	4.6 (2.50–6.62)	0.01	Reference
	Outdoors	221	51	23.1 (17.50–28.64)		6.28 (3.56–11.08)

Age	Young (<6 months)	278	26	9.4 (5.93–12.77)	0.19	Reference
	Adult (≥6 months)	338	43	12.7 (9.17–16.27)		1.41 (0.84–2.37)

Gender	Female	347	38	11.0 (7.67–14.22)	0.82	Reference
	Male	269	31	11.5 (7.70–15.33)		1.06 (0.64–1.75)

Total		616	69	11.2 (8.71–13.69)		

**Table 3:** Variations in bg and gdh gene loci among the subtypes of *Giardia duodenalis* assemblage E in rabbits.

Isolate	Subtype (no.)	Nucleotide at position				GenBank ID
		83	201	429	471	
bg	Ref. sequence	A	A	C	C	KU886048
JT-32	E1 (*n* = 1)	—	—	—	T	MK659571
JT-42	E2 (*n* = 1)	—	G	—	—	MK659572
JT-334	E3 (*n* = 1)	G	—	—	—	MK659573
YT-10	E4 (*n* = 1)	—	—	G	—	MK659574
		24	399			
gdh	Ref. sequence	A	A			KF843962
JT-37	E1 (*n* = 1)	—	G			MK675546
YT-189	E2 (*n* = 1)	C				MK675547

## Data Availability

The *G. duodenalis* prevalence data used to support the findings of this study are included in the article.

## References

[1] Jin Y., Fei J., Cai J. (2017). Multilocus genotyping of *Giardia duodenalis* in Tibetan sheep and yaks in Qinghai, China. *Veterinary Parasitology*.

[2] Feng Y., Xiao L. (2011). Zoonotic potential and molecular epidemiology of *Giardia* species and giardiasis. *Clinical Microbiology Reviews*.

[3] Certad G., Viscogliosi E., Chabé M., Cacciò S. M. (2017). Pathogenic mechanisms of *Cryptosporidium* and *Giardia*. *Trends in Parasitology*.

[4] Yin Y.-L., Zhang H.-J., Yuan Y.-J. (2018). Prevalence and multi-locus genotyping of *Giardia duodenalis* from goats in Shaanxi province, northwestern China. *Acta Tropica*.

[5] Ryan U., Hijjawi N., Feng Y., Xiao L. (2019). *Giardia*: an under-reported foodborne parasite. *International Journal for Parasitology*.

[6] Halliez M. C., Buret A. G. (2013). Extra-intestinal and long term consequences of *Giardia duodenalis* infections. *World Journal of Gastroenterology*.

[7] Squire S. A., Ryan U. (2017). *Cryptosporidium* and *Giardia* in Africa: current and future challenges. *Parasites and Vectors*.

[8] Einarsson E., Ma’ayeh S., Svärd S. G. (2016). An up-date on *Giardia* and giardiasis. *Current Opinion in Microbiology*.

[9] Wang Y., Li N., Guo Y. (2018). Persistent occurrence of *Cryptosporidium hominis* and *Giardia duodenalis* subtypes in a Welfare Institute. *Frontiers in Microbiology*.

[10] Wang X., Cai M., Jiang W. (2017). High genetic diversity of *Giardia duodenalis* assemblage E in pre-weaned dairy calves in Shanghai, China, revealed by multilocus genotyping. *Parasitology Research*.

[11] Zhang X. X., Zheng W. B., Ma J. G. (2016). Occurrence and multilocus genotyping of *Giardia intestinalis* assemblage C and D in farmed raccoon dogs, *Nyctereutes procy*onoides, in China. *Parasites and Vectors*.

[12] Li S., Zeng W., Li R. (2018). Rabbit meat production and processing in China. *Meat Science*.

[13] Yang Z., Zhao W., Shen Y. (2016). Subtyping of *Cryptosporidium cuniculus* and genotyping of *Enterocytozoon bieneusi* in rabbits in two farms in Heilongjiang Province, China. *Parasite*.

[14] Xie X., Bil J., Shantz E., Hammermueller J., Nagy E., Turner P. V. (2017). Prevalence of *lapine rotavirus*, *astrovirus*, and *hepatitis E* virus in Canadian domestic rabbit populations. *Veterinary Microbiology*.

[15] Qi M., Xi J., Li J., Wang H., Ning C., Zhang L. (2015). Prevalence of Zoonotic *Giardia duodenalis* assemblage B and first identification of assemblage E in rabbit fecal samples isolates from central China. *Journal of Eukaryotic Microbiology*.

[16] Pantchev N., Broglia A., Paoletti B. (2014). Occurrence and molecular typing of *Giardia* isolates in pet rabbits, chinchillas, guinea pigs and ferrets collected in Europe during 2006–2012. *Veterinary Record*.

[17] Vasco K., Graham J. P., Trueba G. (2016). Detection of zoonotic enteropathogens in children and domestic animals in a semirural community in Ecuador. *Applied and Environmental Microbiology*.

[18] Jiang J., Ma J.-G., Zhang N.-Z. (2018). Prevalence and risk factors of Giardia duodenalis in domestic rabbbits (*Oryctolagus cuniculus*) in Jilin and Liaoning province, northeastern China. *Journal of Infection and Public Health*.

[19] Zhang X., Qi M., Jing B. (2018). Molecular characterization of *Cryptosporidium* spp., *Giardia duodenalis*, and *Enterocytozoon bieneusi* in rabbits in Xinjiang, China. *Journal of Eukaryotic Microbiology*.

[20] Zhang W., Shen Y., Wang R. (2012). *Cryptosporidium cuniculus* and *Giardia duodenalis* in rabbits: genetic diversity and possible zoonotic transmission. *PLoS One*.

[21] Thompson J. D., Gibson T. J., Plewniak F. (1997). The CLUSTAL_X windows interface: flexible strategies for multiple sequence alignment aided by quality analysis tools. *Nucleic Acids Research*.

[22] Koehler A. V., Haydon S. R., Jex A. R., Gasser R. B. (2016). *Cryptosporidium* and *Giardia taxa* in faecal samples from animals in catchments supplying the city of Melbourne with drinking water (2011 to 2015). *Parasites and Vectors*.

[23] Deng L., Li W., Zhong Z. (2017). Prevalence and molecular characterization of *Giardia intestinalis* in racehorses from the Sichuan province of southwestern China. *PLoS One*.

